# The association of leadership styles and empowerment with nurses’ organizational commitment in an acute health care setting: a cross-sectional study

**DOI:** 10.1186/s12912-016-0161-7

**Published:** 2016-06-09

**Authors:** Samirah A. Asiri, Wesley W. Rohrer, Khaled Al-Surimi, Omar O. Da’ar, Anwar Ahmed

**Affiliations:** Department of Continuous Quality Management and Patient Safety, Armed Forces Hospital, Dhahran, Saudi Arabia; Department of Health Policy and Management, Program Graduate School of Public Health, University of Pittsburgh, Pittsburgh, USA; Department of Health System and Quality Management, College of Public Health and Health Informatics, King Saud bin Abdulaziz University for Health Sciences, Riyadh, Saudi Arabia; Department of Health System and Quality Management, College of Public Health and Health Informatics; King Abdullah International Medical Research Center, King Saud bin Abdulaziz University for Health Sciences, Ministry of National Guard Health Affairs, Riyadh, Saudi Arabia; Department of Epidemiology and Biostatistics, College of Public Health and Health Informatics, King Saud bin Abdulaziz University for Health Sciences, Riyadh, Saudi Arabia

**Keywords:** Transformational leadership, Transactional leadership, Organizational commitment, Psychological empowerment

## Abstract

**Background:**

The current challenges facing healthcare systems, in relation to the shortage of health professionals, necessitates mangers and leaders to learn from different leadership styles and staff empowerment strategies, so as to create a work environment that encourages nursing staff commitment to patients and their organization. This study intends to measure the effects of nurses’ overall perception of the leadership style of their managers, and psychological empowerment on their organizational commitment in acute care units, in National Guard Health Affairs, Riyadh City, Saudi Arabia.

**Methods:**

This was a cross-sectional survey, where the data was obtained from nurses at King Abdulaziz Medical City. Hard copy questionnaires were distributed to 350 randomly selected nurses. Three hundred and thirty two (332) were completed, representing a response rate of 95 %. Three validated survey instruments were used to obtain the data: (1) The Multifactor Leadership Questionnaire (MLQ), formulated by Bass and Avolio (1997), (2) The Psychological Empowerment Scale developed by Spreitzer (1995) and (3) The Three-Component Model of Employee Commitment developed by Meyer and Allen (1997). A theoretical model that conceptually links leadership, empowerment, and organizational commitment was used. The SPSS program version 19 was employed to perform descriptive and inferential statistics including correlation and stepwise multiple regression analysis.

**Results:**

Overall most nurses perceived their immediate nursing managers as not displaying the ideal level of transformational leadership (TFL) behaviors. Nurses’ commitment appeared to be negatively correlated with TFL style and perceived psychological empowerment. However, commitment was positively correlated with the Transactional Leadership (TAL) style. Analysis, also, showed that commitment is significantly associated with the nurse’s nationality by region: North American (*P* = 0.001) and Arab (*p* = 0.027). The other important predictors of commitment include TAL (*P* = 0.027), Laissez-faire Leadership (LFL (*P* = 0.012), and autonomy (*P* = 0.016). The linear combination of these predictors explained 20 % of the variability of the nurses’ commitment.

**Conclusion:**

The study findings suggest that leadership styles and employee empowerment could play an instrumental role in promoting organizational commitment of nurses working in acute health care settings, at least in the Saudi Arabian context.

## Background

Acute care environments in hospitals are in a state of continuous improvement and rapid change due to the pressures of reduced average length of hospital stays (ALOS), cost-containment, unpredictability, and uncertainty. Moreover, the shortage of nursing staff within the hospital increases the difficulty of maintaining acceptable health care standards. Also, by replacing nursing positions, such as the nurse manager, which requires strong leadership and at least minimal management experience and training, with less-qualified health care personnel has led to a devalued nursing role within organizations [1]. Nurses might perceive that managers, both with autocratic and weak leadership styles, present barriers to effective nursing practice. Overly controlling managers are likely to discourage individual initiative and creative thinking. Weak nurse managers, meanwhile, may fail to advocate for nurses’ employment rights, the respect they deserve within the health care team, to support their need for resources, and may fail to effectively negotiate and retain nursing positions. Thus, failed leadership due to the absence of qualified nurse managers results in the nursing staff becoming further disempowered, demotivated and ultimately disengaged, less satisfied and committed [2].

Nursing managers have an essential role in hospital management. This includes facilitating care, ensuring patient safety, enhancing the quality of work life of nurses, and championing change processes that serve these ends [3, 4]. This requires managers to empower their nurses to perform their obligations using best practices. It, also, requires managers to ensure the staffing nurses’ commitment by means of providing an optimal work environment whilst maintaining a high level of quality care and patient safety. Nurse empowerment is not only an essential requirement, but it also affects work performance to achieve these outcomes. In this context, Kanter (1993) states that Work Empowerment Theory is conceptually consistent with the nursing care process and can be logically extended to support nurses’ interactions with their patients, as empowered nurses will enable best care practices for their patients, hence, resulting in optimal patient care outcomes [5]. However, it has been reported that the relations between nurse managers and staffing nurses have grown more strained, leading to less opportunity for nurses to communicate their concerns about patient care and their own roles with their managers [6]. Previous studies showed nurses perceive that they are underrepresented in the organizational hierarchy [4–6]; thus limiting their capacity to have meaningful role in decision making and influencing change to improve organizational processes that are relevant to the nurse’s role, quality of work life and patient care. Correspondingly, these conditions affect not only the staffing nurses’ emotional and physical health outcomes, but also their efficiency, productivity, performance and commitment. Failing to provide nurses with a significant voice in health care system management, eventually leads to adverse effects in the workplace environment and organizational culture and functioning, and this ultimately results in lower-quality patient care [7].

The available literature provides considerable evidence that nurses’ limited participation in clinical decision-making is ineffective and harmful to patient safety. There is evidence that nurses may also be overextending themselves to ensure the quality of care under deteriorating working conditions [5]. It has also been reported that limited participation of nurses in decision-making, affecting their jobs and work environment, entails a cost to the organization in terms of mistrust of hospital management and resentment [6]; high levels of stress, decreased morale, reduced job satisfaction and lower organizational commitment [2, 5, 6, 8, 9]. These conditions collectively would, likely, contribute to nursing burnout and nurses leaving the organization and even the profession.

Thus, as a priority, the role of the nurse manager, in continually and effectively empowering staff in the care process, is to ensure a work environment and culture that encourages and sustains quality of healthcare and patient safety. However, the relationship between leadership style and behavior and employee commitment and empowerment has not been investigated in Saudi Arabian health care context. Furthermore, the work empowerment construct itself has not been previously tested within the Saudi Arabian context. Hence, this study aims primarily to test a model that links leadership style and employee empowerment, and their impact on organizational commitment among nurses working in an acute healthcare setting in Riyadh, Saudi Arabia.

## Methods

### Study design, setting, and sampling

This study uses a cross-sectional survey investigating the relationships among leadership style, psychological empowerment, and organizational commitment. The study was conducted at King Abdulaziz Medical City, Ministry of National Guard Health Affairs, in Riyad, Saudi Arabia (KAMC-R). KAMC-R was established in May 1983 to provide medical, surgical, obstetrician, and critical care services to National Guard employees and their dependents. The services expanded over the following years to include more than 1800 beds with specialized services like oncology and transplant in addition to the original services.

Three hundred and fifty (350) questionnaires were randomly distributed to full time registered nursing staff in the acute care units at KAMC-R. Of these 332 valid questionnaires were completed and returned for data analysis, representing a 95 % response rate.

### Instruments and reliability

Three recognized and validated questionnaires to obtain the data that were used are: the 1997 Multifactor Leadership Questionnaire (MLQ) developed by Bass and Avolio [10], the 1995 Psychological Empowerment Scale developed by Spreitzer [11], and the 1997. Three-Component Model of employee commitment developed by Meyer and Allen [12]. The three instruments address the nurses’ perception of workplace Psychological Empowerment (PE), Leadership Styles (LS), and levels of their Organizational Commitment (OC).

The reliability of the three instruments was investigated in a random sample of 80 nurses. Internal consistency was checked for the 44 items of the LS scale, 12 items of PE scale, and 18 items of the OC scale. Cronbach’s alpha measures calculated were 0.94, 0.94, and 0.79, respectively. Acute care nurses were asked to rate their perceptions of their immediate supervisors’ leadership style, and their own level of commitment to the organization. In addition, demographic profiles including characteristics such as gender, age, nationality education level, years of experience in nursing, and number of years in current position were sought.

### Statistical analyses

**Fig. 1:**
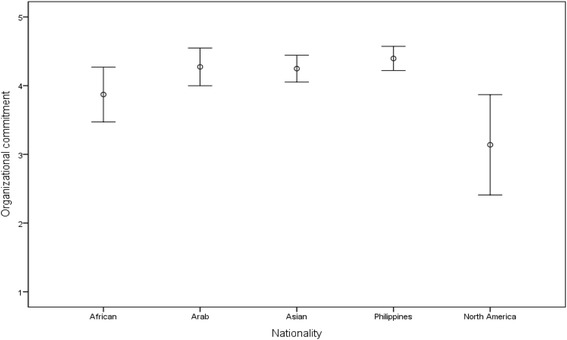
Differences in organizational commitment by nationality: 95 % CI for mean

Data from the completed and returned surveys were compiled and analyzed using the SPSS statistical software package (SPSS for Windows, version 19.0, SPSS, Chicago, IL, USA). First, key variables from the conceptual framework were descriptively summarized. Counts and percentages were used to summarize categorical variables (Table 1). Mean and standard deviation (Mean ± SD) were used to summarize continuous variables (Tables 2, 3, 4 and 5). Then, inferential statistics such as Pearson Correlation and Stepwise regression were performed to make inferences from the study sample to the broader population. ANOVA and post hoc analysis were conducted to examine differences in nurses’ commitment across various nationalities (Table 4 & Fig. 1). Pearson Correlation coefficients were calculated to measure the direction and strength of the linear relationship between nurses’ commitment across leadership and empowerment perception subtypes (Table 6). Stepwise linear regression analysis was employed to identify the main predictors of nurses’ commitment (Table 7). The following predictors were entered into the model: age, gender, nationality (African, Arab, Philippines, American), leadership styles (TFL, TAL, LFL), empowerment (meaning, confidence, autonomy, impact), and additional qualification degree other than nursing.

**Table 1:** Demographic characteristics of study respondents

Variable		N	%
Gender	Male	26	7.9
	Female	305	92.1
Position	Staff nurse	302	92
	Nurse manager	9	2.7
	Others	19	5.8
Education	Diploma/Associate degree	79	25.4
	Baccalaureate degree	225	72.3
	Master degree	7	2.3
Other Degree	Yes	120	43.6
	No	155	56.4
Nationality	African	14	4.4
	Arab	31	9.7
	Asian	45	14.2
	Filipinos	217	68.2
	North American	11	3.5

**Table 2:** Employees’ empowerment scale and subscales (scores lowest = 0 to highest = 6)

Variables	Mean score	SD
Overall score of empowerment	4.70	.90
Subscales scores of empowerment		
- Meaning	5.16	.95
- Confidence	4.92	.97
- Autonomy	4.48	1.13
- Impact	4.21	1.13

**Table 3:** Organizational commitment scale and subscale (scores lowest = 1 to highest = 7)

Variables	Mean score	SD
Overall score commitment	4.32	1.43
Subscales scores of commitment		
- Normative Commitment	4.54	1.06
- Continuance Commitment	4.46	1.11
- Affective Commitment	4.02	3.46

**Table 4:** Multiple comparisons of organizational commitment by nationality

		Mean Difference	SE	Sig.	95 % Confidence Interval	
					Lower Bound	Upper Bound
North America vs.	African	−0.73	0.47	0.532	−2.03	.57
	Arab	−1.13	0.42	0.053	−2.28	.01
	Asian	−1.11	0.40	0.046^a^	−2.20	−.01
	Philippines	−1.26	0.37	0.007^a^	−2.27	−.24

^a^The mean difference is significant at the 0.05 level

**Table 5:** Leadership style scale and subscale scores (lowest = 0 to highest = 4)

Leadership style scale	Mean score	SD
*Transformational (TFL) overall score*	*2.55*	*0.75*
*TFL subscale scores*		
- Inspirational Motivation (IM)	2.65	0.86
- Idealized Influence Attributed (IAII)	2.59	0.81
- Idealized Influence Behavior (IBII)	2.51	0.83
- Intellectual Stimulation (IS)	2.50	0.83
- Individualized Consideration (IC)	2.47	0.83
*Transactional (TAL) overall score*	*2.12*	*0.64*
*TAL subscale scores*		
- Contingent Rewards CR	2.50	0.87
- Management-by-Exception-Active MBEA	2.47	0.83
- Management-by-Exception-Passive, MBEP	1.36	1.21
Laissez-faire *(TFL) overall score*	*1.26*	*1.02*

**Table 6:** Correlation of employees’ empowerment and leadership styles subscales with organizational commitment scale

Independent variables (subscales)		Dependent variable (Commitment)
Subscales of empowerment		
Meaning	Pearson correlation	−.130^a^
	Sig. (2-tailed)	.019
	N	323
Confidence	Pearson correlation	−.015
	Sig. (2-tailed)	.782
	N	323
Autonomy	Pearson correlation	−.069
	Sig. (2-tailed)	.213
	N	323
Impact	Pearson correlation	−.075
	Sig. (2-tailed)	.180
	N	320
Subscales of leadership styles		
TFL	Pearson correlation	−.113^a^
	Sig. (2-tailed)	.045
	N	316
TAL	Pearson correlation	.124^a^
	Sig. (2-tailed)	.028
	N	314
LFT	Pearson correlation	.093
	Sig. (2-tailed)	.103
	N	306

^a^correlation is significant at the .05 level (2-tailed)

**Table 7:** Stepwise linear regression analysis: predictors of nurses’ commitment

Main variable	Variables	B	SE	t	*P*-value
	Intercept	2.90	0.23	12.82	0.001
Leadership styles	TFL	0.14	0.08	1.8	0.074
	TAL	0.22	0.10	2.22	0.027*
	LFL	0.14	0.05	2.54	0.012*
Nationality	African	−0.38	0.21	−1.77	0.078
	Arab	−0.31	0.14	−2.22	0.027*
	American	−0.89	0.25	−3.63	0.001*
Empowerment	Autonomy	0.09	0.04	2.42	0.016*

*N* = 242, *F* (7, 234) = 9.77, *P*-value =0.001, R Squared = 0.226, Adj.R Squared = 0.203, RMSE = 0.624 * Statistically significant at *P* value less than 0.05

## Results

### Respondents’ characteristics

The characteristics of the sampled nurses are summarized in the Table 1. A total of 332 completed questionnaires were analyzed. The demographic data revealed that 305 (92.1 %) of the sample were females. Of the respondents, 120 (43.6 %) had a degree in fields other than nursing. The average age of nurses in the Adult Acute Care Unit was 35 (±SD 7.94 years), while the average age in the Pediatrics Acute Care Unit was 37 (±SD 8.14 years). A considerable majority 217 (68.2 %) of surveyed nurses were from the Philippines. Demographic variables of the participating RNs were further examined according to their areas of practice. Of the 332 nurses surveyed, 229 (69 %) were in Pediatrics Acute Care Unit and 103 (31 %) in Adult Acute Care Unit.

### Psychological empowerment

Based on the psychological empowerment scores measured on a 6-point Likert scale by the Empowerment questionnaire, 83.2 % of the nurses in this study perceived themselves to be at the moderate level psychologically empowered overall. In particular, they rated themselves high in psychological empowerment on the dimension of meaning with an average rating of 5.16 (±0.95 SD), moderate on the dimensions of confidence with an average rating of 4.92 (±0.97SD), and autonomy with an average rating of 4.48 (±1.13 SD), and lower on impact with an average rating of 4.21 (±1.13 SD), (refer to Table 2).

### Organizational commitment (OC)

The overall OC score measured on a 7-point Likert scale by the Organizational Commitment Questionnaire, showed that the nurses perceived themselves to be moderately committed to the organization, with the means of all components being slightly above the scale midpoint of 3.5. The overall mean score on the 8-items sub-scale dealing with RNs’ affective commitment (AC) was 4.02 (±SD 3.45). The overall mean score on the 8-item sub-scale addressing the RNs Continuance Commitment (CC) was 4.46 (±SD 1.11). The overall mean score on the 8-items sub-scale addressing the RNs’ Normative Commitment (NC) was 4.54 (±SD 1.05), (Refer to Table 3). Nurses appear to be most influenced by the NC component of OC, and somewhat less by CC and the AC components.

There was significant organizational commitment difference between the five subgroups of nationality (*F* = 3.47; df = 4303; *p*-value = 0.009). Post-hoc analysis (Tukey’s test) of organizational commitment by nationality showed that nurses from North America had lower commitment scores compared to nurses from Asia (3.1 ± 1.0 vs 4.3 ± 0.7; *p*-value = 0.046) and the Philippines (3.1 ± 1.0 vs 4.4 ± 1.3; *p*-value = 0.007), (refer to Table 4 and Fig. 1).

### Leadership styles

As shown in Table 5, the overall mean scores for perceiving leadership styles were, TFL (2.55 (± SD 0.75), TAL (2.12 (± SD 0.64), and LFL (1.26 (± SD 1.02). The dominant categories within the TFL composite were the IM and IAII with the highest scores of 2.65 ± (SD 0.86) and 2.59 ± (SD 0.81) respectively while the IC and IS showed the lowest mean scores of 2.47 (± SD 0.83 and 2.50 (± SD 0.83) respectively. The TAL contained the leadership components subscales of Contingent Rewards (CR), and Management-by-Exception Active and Passive, (MBEA and MBEP). Among the three components subscales of TAL, the CR sub-scale had the highest mean score (2.5 ± SD 0.87) followed by MBEA (2.47 ± SD 0.83) while the MBEP had the lowest score of 1.36 (± SD 1.21).

### Impact of leadership style and employee empowerment on organizational commitment

As shown in Table 6, the nurses’ commitment was significantly negatively correlated with meaning dimension of Commitment (Pearson Correlation = -0.130, *p*-value = 0.019) and TFL (Pearson Correlation =−0.113, *p*-value = 0.045). On the other hand, nurses’ commitment was significantly positively correlated with TAL (Pearson Correlation = 0.124, *p*-value =0.028). In addition to the correlation and descriptive analysis, we measured the effects of the nurses’ perceptions of leadership style and psychological empowerment on nurses’ commitment and demographic factors. Stepwise, multiple linear regression was performed to identify the best explanatory variables based on the conceptual framework with controlling for leadership and empowerment perception subtypes, as well as the nurse social and demographic profiles (refer to Table 6.)

Table 7 shows the results of the stepwise regression analysis for multiple regression of nurses’ commitment on TFL, TAL, LFL, autonomy, and nationality. Although this method removes the least-significant term to re-estimate effects in subsequent iteration, the researchers decided to include it in the model predictors with a *p*-value less than 0.1.

Finally, the regression analysis showed that the nurses’ perception of leadership styles (TAL and LFL) had positive and significant effects on commitment (*p*-value = 0.027 and 0.012), respectively. For instance, nurses’ commitment tends to increases along with perceived autonomy and leadership styles as would be expected from the conceptual model.

In summary, a notable finding is that perceived of TFL was not significant (*p* = 0.074) compared with other leadership styles. With regard to psychological empowerment subtypes, autonomy appears to affect commitment significantly in a positive direction (*p* = 0.016). Subsequently, when other demographic and nurse characteristic variables were controlled, the analysis revealed that the predicted nurses’ commitment was lower for American nurses by 0.89 (*p*-value =0.001) and Arab nurses by 0.31 (*p*-value =0.027). This suggests that Arab and American nurses tend to have lower commitment relative to nurses of other nationalities. The findings showed that there was lower commitment amongst American nurses compared to Arab nurses, while African nurses were marginally lower in commitment compared to their Arab colleagues. Together, the linear combination of these explanatory variables was significantly related to nurses’ commitment, *F* (7, 234) = 9.77 with *p*-value = 0.0001). The correlation coefficient for this model was 0.48 which indicates there is a moderate association between the observed and the predicted nurses’ commitment. The adjusted R-square was 0.20, indicating that approximately 20 % of the variance of the nurses’ commitment can be accounted for by the linear combination of these explanatory variables. In other words, the variables of TAL, LFL, autonomy, and nationality together predict 20 % of the variance in commitment of nurses in such a health care environment.

## Discussion

The relationship between leadership styles and staff perceptions of their empowerment is important for nursing mangers and leaders, in order to create a work environment that encourages and facilitate a high level of commitment among the nursing staff. This is particularly important, especially in the wake of the current challenges facing healthcare systems in relation to the shortages of health professionals, especially among nursing profession.

In our study, nurses perceive themselves to have moderate levels of overall psychological empowerment, high levels of meaning, moderate levels of confidence and autonomy, and low impact on their working environment. These results are consistent with findings from other previous studies in acute care settings [2, 4, 6, 13]. These findings suggest nurses may feel less motivated when they perceive that their work has no significant value to the organization, and important aspects of their job, work environment, and patient care. This may be experienced as burnout or apathy and the nurses’ sense that they are exerting too much effort with too little reward or return.

The psychological empowerment subscale, autonomy, was the only statistically significant predictor of commitment, suggesting that nurse managers have and authentic commitment to full engagement of the nurses in appropriate decision-making about patient care processes, patient safety and their working environment. This finding is inconsistent with literature showing that increased participation empowerment facilitates greater commitment. In this context, Clifford (1992) noted that if management does not allow for staff participation in decision-making, the effort to empower frustrates employees, resulting in an increased dependence on authoritarian structures. The findings further show that AC has the lowest mean score, where nurses who did not feel a sense of belonging or attachment to their organization are less likely to stay with their organization to find more desirable jobs than nurses with high AC. This result is inconsistent with existing literature [12, 14–16]. Thus, the development of AC is influenced by the degree to which an organization shows that it values and supports its employees. In contrast, NC has the highest mean score, suggesting that some nurses are staying with the organization because of a sense of obligation and moral responsibility not because they “want to” based on other factors, for example, the perception that their work is appreciated. This particular result is consistent with literature showing that feeling of obligation to stay with an organization probably results from internalization of normative pressure exerted on an individual before entry into the organization (i.e. cultural socialization, or familial) or following entry (i.e. organization socialization) [17].

The study results indicate that Acute Care nurse managers are perceived by nurses to exhibit some elements of TFL behavior. However, the total scores for the TFL and TAL leadership subscales are less than what have been studied by Bass and Avolio (1997), and which they consider to be ideal levels for *effective leadership and* suggest that the mean scores for the most effective leadership should be greater or equal to 3.0 or TFL components. This benchmark shows that nurse managers who have a mean score greater than 3.0 are more likely to achieve the desired outcomes than low-rated TFL managers. According to our study findings, the nurse managers in this environment can be characterized as having a moderate level of effective TFL based on the perceptions of the nurses surveyed. Thus, the study results suggest that nurse managers tend to focus more on nurse compliance and task completion, emphasize assignments, work standards, and task-oriented goals, and depend more on organizational punishments and rewards to influence nurses’ performance.

Contrary to expectation that TFL promotes employees’ capacities and capability, the results of the study further reveal that nursing managers in acute care units do not motivate and encourage their nurses to envision attractive future states as indicated by IM mean score of 2.65 (SD = 0.86). These findings is inconsistent with studies showing that employees who have high level of capabilities feel a greater sense of control and are more likely to try learning more to become even more capable (Avolio & Bass, 1995). The nurses in our study rated Individualized Consideration (IC) with a lower score implying that nurse managers often do not display IC leadership behaviors in interaction with their staffing nurses. According to our conceptual model, these managers should pay more attention to the nurses’ self-efficacy and their organizational performance. Taking the initiative to interact with their employees and responding appropriately to personal concerns and needs expressed by their staff should help them to be more effective and trusted managers [18]. Accordingly, the leadership training for nursing managers should emphasize supportive and responsive leadership to support nurses and encourage their autonomy and empower them to take on more accountability in line with their growing expertise and engagement [19].

Overall, our study findings suggest that the area where the most improvement is required to develop TFL behavior is *Intellectual Stimulation (IS),* the aspect of leadership that was rated with the lowest among the nurses surveyed. In order for IS to be effectively cultivated and nurtured as a way of life in the health care organizations. The “best and brightest” nurse managers should be hired, developed, and rewarded. In addition, creativity and innovation must be fostered in the acute nursing care units by allowing individuals to perform specialized and more challenging work in order to grow their talents and creativity. In turn, this should increase the self-confidence of nurses in performing their work and developing their competencies. Nurse managers should be trained to teach their nurses to see challenges as opportunity for improvements and to encourage them to identify and test new methods and ways of looking at and resolving old problems and, also, to value novel and diverse perspectives.

Moreover, the nursing managers are advised to demonstrate behaviors that will encourage the development of mutual trust, respect, and competence, which will increase the credibility and authority of the nurse manager and provide inspirational and normative behavior. Although not all nurse managers can be expected to possess charismatic personalities, the dimensions of transformational leadership discussed should encourage followers to respect and admire their leaders as role models and valued advocates. Thus, health care organizations should recruit and promote nurse managers who possess these transformational qualities, who are likely to be more effective in instilling shared mission, vision, and values as guidelines for achieving organizational goals. Nurse Managers should promote their organization by demonstrating transformational leadership traits as models for the nursing staff. Furthermore, policy makers, health care educators, and executives should insure that nursing education and supervisory training includes the development of transformational leadership qualities among the nurse managers.

The second leadership style (TAL) contains the leadership components of *Contingent Rewards*, and *Management*-*by*-*Exception* both *Active and Passive forms*. According to Bass et al. (2003) [20], the TAL style is an essential precondition for TFL as it helps to ground the relationship between the follower and the leader. Among the three components of TAL, the *contingent reward CR* subscale had the highest mean score among the nurses surveyed which is consistent with the findings from other studies [21]. Consequently, this result indicates that nurse managers often structure and clarify the task requirements and roles for their followers. They discuss performance expectations and outcomes with nurses; explain how these results are achieved, and the rewards they should expect for their performance and satisfactory effort. In general, these nurse managers provided tangible and intangible resources and support to followers in exchange for the nurses’ efforts and contributions. In this context, Bass and Avolio (2003) stated that the ideal CR rating must be *greater* than the benchmark rating of 2. In our study, the mean score of the nurses’ responses to CR was 2.50, which satisfies this criterion. On the other hand, Bass and Avolio (2003) indicate that the mean score of MBEP should be *less* than 1 score and the rate of MBEA subscale should be *less* than 1.5 for effective managers. In our study findings, the mean score for MBEA was 2.47, which is outside the desired range indicating that nurse managers actively keep track of all mistakes, concentrate on enforcing standards, and directly monitor staff behavior and performance [22, 23].

In our study, the mean MBEP rating of 1.36 exceeds the benchmarks set by Bass and Avolio (2003) as ideal for a leader; this result suggests that these nurses perceive their nurse managers as not effective in taking corrective action or resolving problems. This implies that the acute care nurse managers tend to wait for problems and mistakes to occur before taking any proactive action [20], and interfere only when the minimum standards have not been met [24].

The Management-by-Exception style*, Active* and Passive forms, emphasizes the controlling aspects of management, where leaders intervene only when things go wrong (Bass et al., 2003). Criticism, correction, negative contingent reinforcement, and negative feedback are examples of interventions that nurse managers rely on in managing-by-exception. Both active and passive forms of the Management-by-Exception style use more negative than positive reinforcement, a pattern associated with low satisfaction with leaders by their followers [25]. Subsequently, it is clear that appropriate supervisory training and reinforcement must be provided for nurse managers to learn and sustain more effective leadership skills.

In summary, the findings of our study indicate that the TFL style has moderate positive association with organizational commitment (OC) and were found to have relationship with both Continuance and Normative forms of commitments. This suggests that TFL leadership style affects how employees feel about their relationships with their managers and their desire and obligation to maintain their employment in the organization. Furthermore, the TAL style was found to have a strong positive relationship with perceived empowerment. These results suggest that managers accorded high TAL scores are those who have achieved high level of trust among their employees, and who in turn delegate maximum authority and responsibility to their followers. As a result, of such leadership, nurses feel they are empowered and are more committed to organizational outcomes. Our findings suggest that a TFL style can achieve a high level of employee commitment through empowerment strategies and meaningful participation in decision-making. The high TFL manager focuses on empowering their employees by delegating power to them and involving them in decision making which in turn leads to a higher level of commitment. This becomes a virtuous circle in terms of the retention of high performing and empowered nurses and successful nurse managers.

### Strengths, limitations, and areas for further research

It is our understanding that this is the first study of its kind, studying the relationships among nursing leadership styles, nurse’s empowerment, and commitment that has been conducted within the healthcare settings in Saudi Arabia. The strength of this study is the focus on nurses and aggregate perception of these relationships, revealing the texture of their day-to-day experiences with their managers and the effect of their sense of empowerment and commitment. However, the findings of this study are limited to a population of acute care nurses within a single health care institution in the Saudi context. Thus, the replication of this study to *different* units and settings within Saudi Arabia and the Gulf Region and Eastern Mediterranean will be necessary and instructive to test whether these findings can be generalized to all nurses in this region and beyond.

Future research should be designed to focus on other factors that might contribute to the level of commitment and empowerment among nurses in other national contexts and organizational environments. Furthermore, special attention should be given to investigation of the factors that can influence retention of talented, highly committed, and empowered nurses. This is of special current relevance within Saudi Arabia given the enforcement of a national labor policy leading to greater Saudization of the professional work force. This could have profound implications for Saudi women, as nursing is one of the relatively few venues for professional work currently available to them.

### Implications of the study

The current study builds on existing leadership literature by providing further evidence for the positive effects of leadership styles on followers, (Bass and Avolio, 1991), and specifically the relationship of TFL to employee empowerment and organizational commitment. Therefore, the findings of this study contribute to the existing literature on the effect of leadership style on nursing practice and outcomes. Further studies could evaluate the benefits, challenges, and financial implications of developing innovative leadership styles in meeting today’s changing health care environment, especially within the Middle East and Gulf Region. The results of this study can be used to better inform decision makers wanting to influence the nursing shortage. These results indicate nursing leaders in hospital settings can enhance the work environment and increase retention by increasing satisfaction for all nurses by training and rewarding managers to adopt transformational leadership behaviors. Other findings of the study suggest the central role of empowerment in the relationship between leadership style and organizational commitment. Evidence supports that the TFL style can foster employee’s commitment through empowerment strategies. The TFL-oriented leader focuses on empowering employees in the workplace, by delegating power to subordinates and involving them in decision making, which in turn leads to increased level of commitment to the organization.

Given the challenges facing the health care system globally, nurse managers will be required to learn new leadership competencies to create an empowered work environment. As demonstrated by this study, leadership style should play an important role in increasing staff nurses’ desire to work with the organization because they “truly want to” and not because they “have to”. Such leadership enhances the meaningfulness of work, encourages the nurse’s participation in decision-making that impacts on her work life and culture and encourages the full engagement of nurses in ensuring patient safety and providing health care of the highest quality.

## Conclusion

The main objective of this study was to test a conceptual framework, relating leadership styles of managers to nurses’ perception of empowerment and their level of commitment. The results of this study show that the nurses surveyed report a lower level of AC relative to NC and CC, suggesting that these nurses do not feel a strong sense of belonging or attachment to their organization. Leadership styles and psychological empowerment significantly affect commitment levels. Other factors being equal, the Transformational, Transactional and Laissez-faire styles positively affect commitment levels, with the Transformational style having a marginal effect. Similarly, the results reveal that autonomy is the only component of the psychological empowerment construct that significantly affects commitment levels. Finally, the results reveal that Arab national and North American nurses report lower commitment levels in relation to nurses of other nationalities.

Overall the findings suggest that nursing leaders in hospital acute settings can enhance the nursing work environment by practicing appropriate leadership styles and empowering strategies, including greater participation of nursing staff in the decision making process. Ultimately, more effective nursing management should result in improved nursing staff retention, job satisfaction, and work commitment.

## Abbreviations

AC, Affective Commitment; ALOS, Average Length of Hospital Stays; CC, Continuance Commitment; CR, Contingent Reward; IAII, Idealized Influence Attributed; IBII, Idealized Influence Behavior; IC, Individualized Consideration; IM, Inspirational Motivation; IS, Intellectual Stimulations; LFL, Laissez-Faire Leadership; MBEA, Management-By-Exception-Active; MBEP, Management-By-Exception-Passive; MLQ, Multifactor Leadership Questionnaire; NC, Normative Commitment; NGAH, National Guard Health Affairs; OC, Organizational Commitment; TAL, Transactional Leadership; TFL, Transformational Leadership
